# Influence of Epstein–Barr virus and human papillomavirus infection on macrophage migration inhibitory factor and macrophage polarization in nasopharyngeal carcinoma

**DOI:** 10.1186/s12885-021-08675-x

**Published:** 2021-08-18

**Authors:** Guofei Feng, Yifei Xu, Ning Ma, Kaoru Midorikawa, Shinji Oikawa, Hatasu Kobayashi, Satoshi Nakamura, Hajime Ishinaga, Zhe Zhang, Guangwu Huang, Kazuhiko Takeuchi, Mariko Murata

**Affiliations:** 1 Department of Environmental and Molecular Medicine, Mie University Graduate School of Medicine, Tsu, 514-8507 Japan; 2 Department of Otorhinolaryngology-Head and Neck Surgery, Mie University Graduate School of Medicine, Tsu, 514-8507 Japan; 3 Department of Otorhinolaryngology-Head and Neck Surgery, First Affiliated Hospital of Guangxi Medical University, Nanning, 530021 China; 4 Graduate School of Health Science, Suzuka University of Medical Science, Suzuka, 510-0226 Japan; 5 Key Laboratory of High-Incidence-Tumor Prevention & Treatment, Guangxi Medical University, Nanning, 530021 China

**Keywords:** Nasopharyngeal carcinoma, Virus, Tumor-associated macrophages

## Abstract

**Background:**

To assess the effects of Epstein–Barr virus (EBV) and human papillomavirus (HPV) infection on the tumor microenvironment, we examined the relationship between viral infection status, macrophage migration inhibitory factor (MIF), and tumor-associated macrophages in nasopharyngeal carcinoma (NPC).

**Methods:**

A tissue microarray containing 150 cores from 90 patients with NPC and six with chronic inflammation was used. EBV and HPV status were detected using in situ hybridization with commercial EBER1 and HPV16/18 probes. Immunofluorescence double staining of MIF, pan-macrophage marker CD68, M1 macrophage marker CD11c, and M2 macrophage marker CD163 were analyzed using the same tissue microarray. The levels of these markers between NPC and inflammation cases and between tumor nests and stroma were compared. Correlations among these markers were analyzed.

**Results:**

We found EBER1(+) cases in 90% of NPC patients, including 10% EBV/HPV co-infection. M1 macrophages mainly infiltrated the tumor nest, while M2 macrophages infiltrated the tumor stroma. We found a significant positive correlation between EBER1 levels and MIF levels in tumor nests and a significant positive correlation between HPV16/18 and CD11c(+) cell levels in NPC tissues.

**Conclusions:**

It is suggested that MIF is associated with EBV, and M1 macrophage infiltration is affected by HPV status in NPC.

**Supplementary Information:**

The online version contains supplementary material available at 10.1186/s12885-021-08675-x.

## Background

Nasopharyngeal carcinoma (NPC) is a malignant epithelial tumor that originates from the nasopharyngeal mucosal lining. It is rare worldwide; however, prevalent in southern China and Southern Asia [1]. Approximately 70% of patients with NPC are diagnosed at an advanced stage and are more prone to therapy failure due to radioresistance [2]. NPC is characterized by Epstein–Barr virus (EBV) infection and substantial lymphocyte infiltration [3], which consists of the immune microenvironment, indicating the importance of EBV and the tumor microenvironment in NPC pathogenesis. EBV infection is considered the most important etiological factor of NPC [4] and is reported to change the tumor microenvironment to benefit itself, particularly immune evasion [5]. As EBV and human papillomavirus (HPV) are two common viruses in head and neck cancer, the influence of HPV on NPC should be clarified. Despite being an important component of the tumor microenvironment, the relationship among tumor-associated macrophages (TAMs), EBV, and HPV infection remains unknown in NPC.

It is well-known that TAMs play a significant role in promoting tumor progression and resistance to chemotherapy and radiotherapy [6]. In response to different microenvironments, macrophages can be polarized into two activated phenotypes: “classically activated” M1-like macrophages (stimulated by Toll-like receptor ligands and interferon-γ) and “alternatively activated” M2-like macrophages (stimulated by interleukin (IL)-4/IL-13) [7]. The number of TAMs and the activation status of TAMs are tumor-biology-dependent [8]. M1 macrophages are known to play a pro-inflammatory role, which activates type 1 helper T cells to exert tumoricidal activity against pathogens. M2 macrophages are characterized as anti-inflammatory macrophages and are involved in the promotion of angiogenesis and tumor progression [9]. Currently, some macrophage-related markers are available for research on TAMs. CD68 is a glycosylated type I transmembrane protein that is used as a pan-macrophage marker to identify both M1 and M2 macrophages [10]. The CD11c antigen is indicative of M1 polarized macrophages [11]. CD163 is a scavenger receptor and a specific marker of M2 polarized macrophages [12]. It is important to investigate the factors that affect macrophage polarization in cancer.

Macrophage migration inhibitory factor (MIF) is a pro-inflammatory cytokine secreted by inflammatory cells that acts as a strong inhibitor of the random movement of macrophages [13]. MIF is involved in the progression, invasion, and proliferation of many cancers [14]. A recent study suggested that MIF plays a dominant role in M2 macrophage functional polarization, and MIF deficiency spontaneously reverts to an M1-like polarization [15]. It has been reported that MIF was upregulated in NPC and was related to a lower survival rate [16]. However, the effect of MIF on macrophage polarization in NPC remains unclear.

The present study examined EBV and HPV infection status in NPC tissues. Furthermore, we investigated the relationship between viral infection, MIF, and macrophage infiltration in NPC.

## Materials and methods

### Public microarray data

NPC microarray data of GSE13597, GSE12452, and GSE53819 containing NPC tissues and normal nasopharyngeal epithelium (NNE) tissues, were obtained from The Gene Expression Omnibus (GEO) Database (http://www.ncbi.nlm.nih.gov/geo/). The level of MIF between NPC and NNE was analyzed by GEO2R tools.

### Tissue microarray

An NPC tissue microarray (TMA_NPC1504) of 150 cores, containing six (six in duplicates) cases of chronic inflammation and 90 (48 in duplicates) cases of NPC, were purchased from the US Biomax Company (Rockville, MD, USA). To compare different markers in the same tissue, three serial cut slides were used on this tissue microarray with a thickness of 4 μm. The six chronic inflammation cases included four males and two females with a mean age of 47.7 years. The 90 NPC cases included 63 men and 27 women with a mean age of 48.5 years, all of which were undifferentiated non-keratinizing carcinomas.

### In situ hybridization of EBV-encoded RNA (EBER) and HPV RNA

The RNAscope® 2.5 HD Duplex Reagent Kit (Cat. No. 322430, Advanced Cell Diagnostics, Newark, NJ, USA) was used to determine EBV and HPV status on the same tissue microarray. EBV and HPV statuses were detected using a commercial EBER1 probe (Cat. No. 310271) marked with red and HPV16/18 (type 16 and 18, pooled) probes (Cat. No. 311121), marked in green. Human HeLa cell pellets were used as control slides and detected using two ACD positive control probes (POLR2A marked red and PPIB marked green) and a negative control probe (DapB) separately, for quality control. The RNAscope assay was performed according to RNAscope 2.5HD duplex detection for formalin-fixed paraffin-embedded (FFPE) tissues according to the manufacturer’s protocol.

The probe signals were examined under a bright-field microscope (BX53, Olympus, Tokyo, Japan) at a magnification of 400×. The staining results were categorized into five grades according to the number of dots per cell and the number of positive cells, as suggested by the ACD manufacturer. Zero scores (no staining or less than 1 dot in every 10 cells), 1 (1–3 dots/cell), 2 (4–10 dots/cell with very few dot clusters), 3 (> 10 dots/cell with less than 10% positive cells having dot clusters), and 4 (> 10 dots/cell with more than 10% positive cells having dot clusters). Representative image of each score is shown in Additional file 1: Fig. S1.

### Immunofluorescence double staining

FFPE NPC tissue microarray sections were routinely deparaffinized, hydrated, antigen retrieved, and blocked. The sections were then incubated with two sets of primary antibodies at room temperature overnight: a double staining set of rabbit anti-MIF antibody (1:100 dilution, a kind gift from Dr. Takuma Kato, Mie University) and mouse anti-CD68 antibody (1:100 dilution, Cat. No. sc20060, Santa Cruz Biotechnology, Dallas, TX, USA) and a double-staining set of rabbit anti-CD11c antibody (1:100; Cat. No. ab52632; Abcam, Cambridge, UK), and mouse anti-CD163 antibody (1:100; Cat. No. ab156769, Abcam). Then, the slides were incubated using a mixed solution of the secondary antibodies Alexa Fluor Plus 488 goat anti-mouse IgG (1:400 dilution, Cat. No. A32723, ThermoFisher, Waltham, MA, USA), and Alexa Fluor Plus 594 goat anti-rabbit IgG (1:400, Cat. No. A32740, ThermoFisher) for 1.5 h in the dark. Finally, the slides were mounted using DAPI Fluoromount-G (Cat. No. 0100–20, SouthernBiotech, Birmingham, AL, USA) for nuclear counterstaining. The staining results were observed and photographed using an inverted fluorescence microscope 24 h after mounting.

The images were assessed by two investigators. Epithelium and non-epithelium in inflammation tissues, tumor nests, and tumor stroma in NPC tissues were assessed separately. MIF scores were assessed according to staining intensity as 0 (negative), 1 (weak), 2 (moderate), 3 (strong), and 4 (very strong). Representative image of the score is shown in Additional file 2: Fig. S2.

The expression of CD68, CD11c, and CD163 was determined by the average number of positive macrophages as 0 (< 10), 1 (10–39), 2 (40–69), 3 (70–100), and 4 (> 100) in three high magnification fields (400X). Only cells with macrophage-like morphology and expression on the cell membrane were considered as positively stained. Representative image of each score is shown in Additional files 3, 4 and 5: Fig. S3–S5.

### Statistical analyses

Statistical analyses were performed using the SPSS software 20.0. The staining scores between the tumor nest and matched tumor stroma in NPC tissues and the staining score for MIF between the epithelium and non-epithelium area in inflammation tissues were assessed using the Wilcoxon signed-rank test. The differences between inflammation tissues and NPC tissues were assessed using the Mann-Whitney U test. The correlations among the scores for viral infection (EBER1 and HPV16/18), MIF, and macrophage markers (CD68, CD11c, and CD163) were analyzed using Spearman’s correlation test. Statistical significance was set at *p* < 0.05.

## Results

### EBER1 and HPV16/18 infection status in NPC and inflammation cases

After in situ hybridization (ISH), 79 NPC cases and five control inflammation cases were available in TMA_NPC1504, and 12 cases were lost during the ISH procedure. As shown in Table 1, 71 of 79 (90%) patients with NPC were EBER-positive (EBER1(+)), and eight of 79 (10%) patients were HPV16/18-positive (HPV16/18(+)). These eight cases were HPV16/18 and EBER1 double-positive. All five control inflammation cases were both EBER1- and HPV16/18-negative. ISH revealed that EBV and HPV co-infections were observed in 10% of 79 NPC patients.

**Table 1:** NPC and inflammation cases with various EBV and HPV statuses

Type		HPV16/18(+)^**a**^	HPV16/18(−)^**b**^
		N (%)	N (%)
**NPC (*n*** **= 79)**	**EBER1(+)**	8 (10.1)	63 (79.7)
	**EBER1(−)**	0	8 (10.1)
**Inflammation (*n*** **= 5)**	**EBER1(−)**	0	5 (100)

^**a**^ (+) means scores > 0; ^**b**^(−) means scores = 0, *NPC* Nasopharyngeal carcinoma, *EBV* Epstein–Barr virus, *HPV* Human papillomavirus

### MIF, CD68, CD11c, and CD163 scores in inflammation and NPC tissues

To validate the levels of these markers in inflammation and NPC tissues, MIF and CD68 were stained in one tissue microarray slide, and CD11c and CD163 in another tissue microarray slide, using immunofluorescence double staining. After removing tissue lost cases and poorly stained cases, the remaining were used for analysis.

**Fig. 1:**
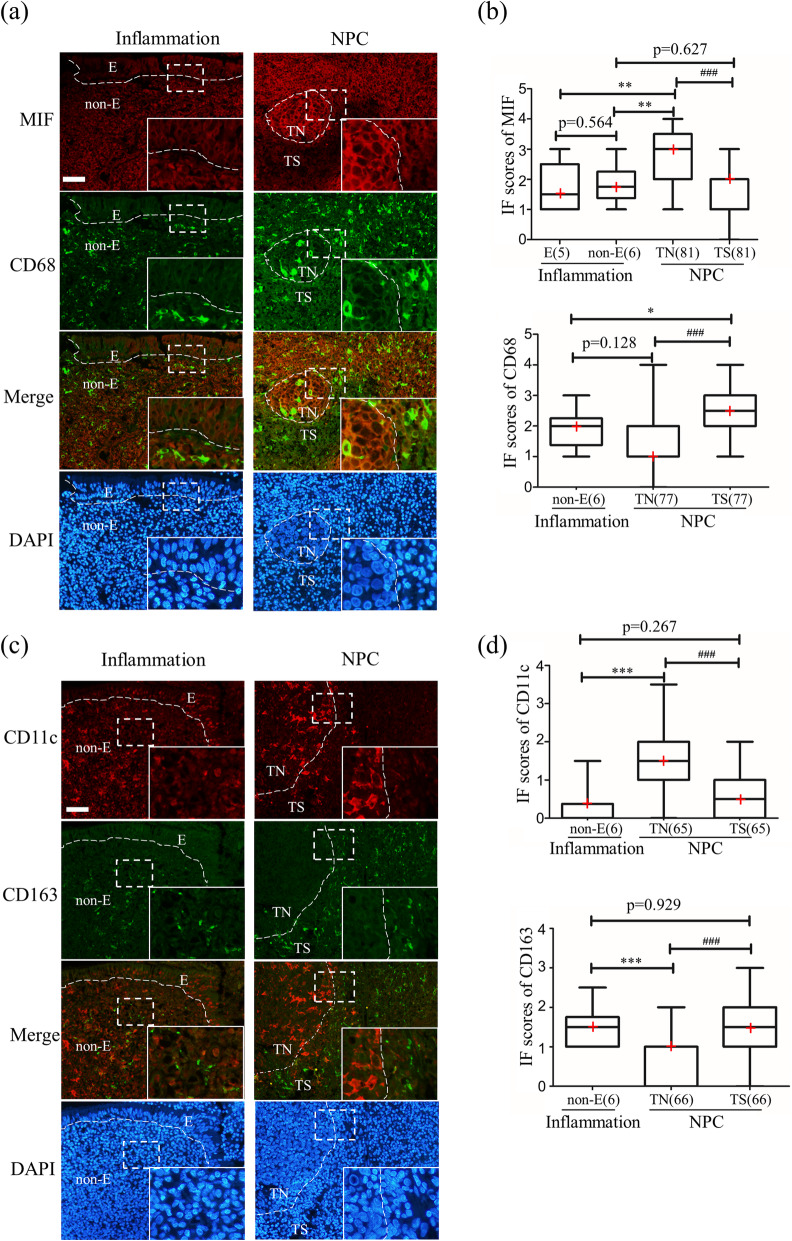
Immunofluorescence of macrophage migration inhibitory factor (MIF) and macrophage markers in NPC tissues. (**a**) Representative images of the inflammation and NPC images of MIF (red), CD68 (green), merged images of the red and green channels, and nuclei counterstained using DAPI. (**b**) Graphic presentation of the IF scores for MIF and CD68. (**c**) Representative images of CD11c (red), CD163 (green), merged images, and DAPI. (**d**) Graphic presentation of IF scores for CD11c and CD163. “E” indicates epithelia, “non-E” indicates the non-epithelial area, “TN” indicates tumor nest, “TS” indicates tumor stroma. The red cross in the graphic represents the median. *: *p* < 0.05, **: *p* < 0.01, ***: *p* < 0.001 analyzed by unpaired test, ###: p < 0.001 analyzed by paired test. An enlarged image is shown in the inset. Scale bar represents 50 μm

MIF immunoreactivity was moderately observed in both epithelial and non-epithelial areas, strongly observed in tumor nests, and moderately observed in tumor stroma (Fig. 1a). The MIF score in the tumor nest was significantly higher than that in the tumor stroma of the NPC tissues (Fig. 1b). The MIF score of NPC cells in the tumor nest was significantly higher than those of the epithelial and non-epithelial tissues of inflammation cases. There was no significant difference in MIF scores between the epithelial and non-epithelial tissues of inflammation cases, and no significant difference was observed between non-epithelial tissues of inflammation cases and tumor stroma. We further screened out three GEO datasets and confirmed that the mRNA level of MIF was also significantly up-regulated in NPC tissues than normal tissues (Additional file 6: Fig. S6).

For CD68 pan-macrophage marker, some CD68-positive (CD68(+)) cells were observed in non-epithelial areas and tumor nests, and many CD68(+) cells were observed in the tumor stroma (Fig. 1a). The score for CD68(+) cells in tumor stroma was significantly higher than that in tumor nest and non-epithelium areas of inflammation tissues, suggesting that there was more macrophage infiltration in tumor stroma than in tumor nests, as well as in non-epithelial areas of inflammation cases (Fig. 1b).

For the M1 marker, some CD11c-positive (CD11c(+)) cells were observed in the non-epithelial area, several CD11c(+) cells were observed in the tumor stroma, and many CD11c(+) cells were observed in the tumor nest. For the M2 macrophage marker, several CD163-positive (CD163(+)) cells were observed in non-epithelial areas and tumor nests, and many CD163(+) cells were observed in the tumor stroma (Fig. 1c). Tumor nests showed significantly higher scores for CD11c(+) cells than in tumor stroma, while the score for CD163(+) cells was significantly higher in tumor stroma than in tumor nests (Fig. 1d). This indicates that there were more M1 macrophages in the tumor nest and M2 macrophages in the tumor stroma of NPC tissues. We also found a significantly higher score for CD11c(+) cells and a lower score for CD163(+) cells in the tumor nest than those in the non-epithelial area of inflamed tissues (Fig. 1d).

### Correlation of MIF, CD68, CD11c, and CD163 with EBER1 and HPV16/18 levels

**Fig. 2:**
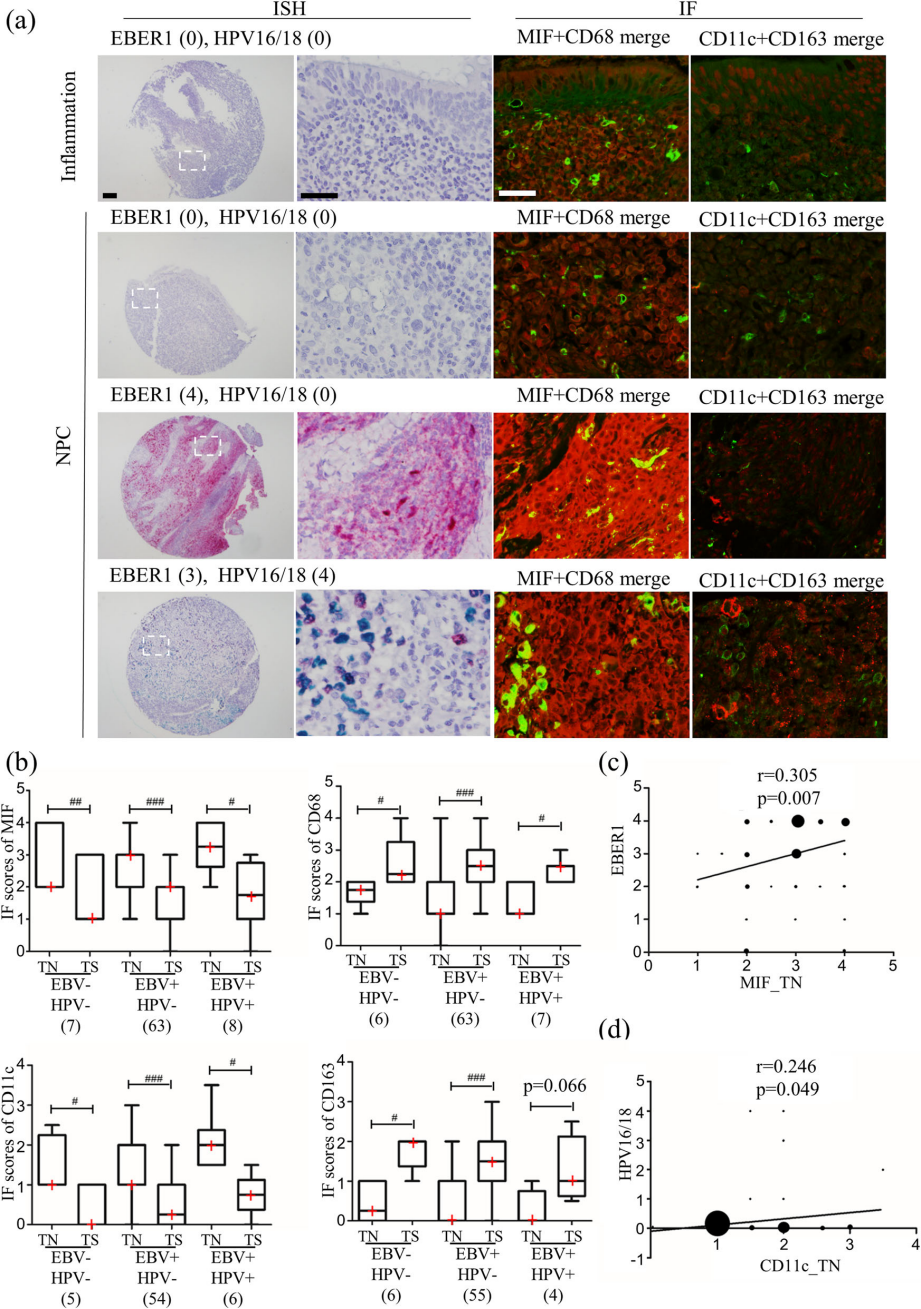
Correlation between virus infection status and macrophage migration inhibitory factor (MIF), macrophage markers. (**a**) In situ hybridization (ISH) images of EBER1 (red) and HPV16/18 (green), and immunofluorescence (IF) images of MIF (red) and macrophage markers, CD68 (green), CD11c (red), and CD163 (green). (**b**) Graphic presentation of the IF scores for MIF, CD68, CD11c, and CD163 in subgroups by EBV and HPV status. “TN” indicates tumor nest, “TS” indicates tumor stroma. #: p < 0.05, ##: p < 0.01, ##: p < 0.001, analyzed by paired test. The red cross in the graphic represents the median**.** (**c**) Correlation between EBER1 level and MIF score in tumor nest among 78 nasopharyngeal carcinoma (NPC) cases. (**d**) Correlation between HPV16/18 level and CD11c score in tumor nest among 65 NPC cases. The size of the dot represented the number of cases. For ISH low magnificent image, the scale bar represents 100 μm, and the other scale bar represents 40 μm

Figure 2a shows ISH images of EBER1 and HPV16/18 with corresponding IF staining of MIF and macrophage markers. In the EBER1-positive NPC case, higher expression of MIF was observed compared to EBER1-negative NPC cases. In the HPV16/18-positive NPC case, a higher score for CD11c was detected compared to the HPV16/18-negative NPC case. We divided NPC patients into three subgroups based on EBV and HPV status (EBV−/HPV-, EBV+/HPV-, EBV+/HPV+) and investigated MIF, CD11c, CD68, and CD163 IF scores in tumor nest and tumor stroma. Significant differences of IF scores between tumor nest and tumor stroma in NPC subgroups were observed (Fig. 2b), similar to NPC group (Fig. 1). Although there was no statistical significance between NPC subgroups by using Kruskal-Wallis test, MIF scores in tumor nest showed the trend as EBV+/HPV+ > EBV+/HPV- > EBV−/HPV-. Also, CD11c IF scores in tumor nest showed the trend as: EBV+/HPV+ > EBV+/HPV- ≈ EBV−/HPV-. The infection of both EBV and HPV may have some influence on MIF and macrophage polarization. Furthermore, we investigated the correlation between viral infection status (EBER1 and HPV16/18), MIF, and macrophage markers (CD68, CD11c, and CD163) in NPC cases using Spearman’s correlation test (Table 2). We found a significant positive correlation between EBER1 levels and MIF expression levels in tumor nests (r = 0.305, *p* = 0.007, Fig. 2c). Also, we found a significant positive correlation between HPV16/18 levels and the score for CD11c(+) cells (r = 0.246, *p* = 0.049, Fig. 2d). On the other hand, the correlations of MIF scores with CD68, CD11c and CD163 scores in tumor nest and tumor stroma of NPC patients showed no statistical significance (Additional file 7: Table S1), suggesting that MIF may not directly affect to CD68, CD11c and CD163 in NPC.

**Table 2:** Correlation analysis among virus infection status and MIF, macrophage markers in NPC patients

Spearman correlation		MIF		CD68		CD11c		CD163	
		TN	TS	TN	TS	TN	TS	TN	TS
**EBER1**	r	**0.305**	0.139	−0.136	−0.177	−0.135	−0.218	− 0.058	−0.152
	p	**0.007**	0.226	0.241	0.126	0.285	0.082	0.648	0.227
	n	78	78	76	76	65	65	65	65
**HPV16/18**	r	0.171	0.021	−0.061	−0.123	**0.246**	0.160	−0.081	− 0.126
	p	0.134	0.857	0.599	0.290	**0.049**	0.203	0.523	0.319
	n	78	78	76	76	65	65	65	65

*TN* tumor nest, *TS* tumor stroma, *r* Spearman’s correlation coefficient, *MIF* macrophage migration inhibitory factor, *NPC* nasopharyngeal carcinoma

## Discussion

In our study, EBV(+) cases were observed in 90% of patients with NPC, including EBV/HPV co-infection (10% of NPC patients) using ISH. This was in concordance with a previous study in which co-infection with HPV and EBV was found in 10% of patients with NPC [17]. It was reported that the HPV infection situation in NPC was different in endemic areas and non-endemic areas. In endemic areas, where type II (differentiated non-keratinizing carcinoma) and type III (undifferentiated non-keratinizing carcinoma) mainly existed, the prevalence of HPV(+) was relatively low (7.7%), and HPV(+)/EBV(−) patients showed better prognosis after radiotherapy in Southern China [18]. In non-endemic areas, where type I (keratinizing squamous cell carcinoma) exists to a relatively high extent, HPV infection is frequently found (30%), and HPV(+) and patients with EBV(−)/HPV(−) NPC had worse outcomes than those with EBV(+) NPC [19]. Controversially, some studies have found that HPV did not influence NPC carcinogenesis [20, 21]. To date regarding the interaction between EBV and HPV, it is known that EBV latent gene product latent membrane protein 1 can block p16 expression [22]. For the tissue microarray used in this study, TNM grade for patients with NPC was grade III, and therefore, we could not assess the effect of co-infection on NPC progression. More studies are needed to investigate the role of HPV in NPC pathogenesis.

Our results showed that MIF expression in tumor nests was higher than those in the tumor stroma of NPC and inflammation tissues. Higher expression of MIF has been reported in different cancers, including endometrial cancer, lung adenocarcinoma, hepatocellular carcinoma, colon cancer, and NPC [23–27]. Increased MIF has been demonstrated to be associated with poor survival in colorectal cancer, oral squamous cell carcinoma, gastric cancer, and NPC [16, 28–30]. Furthermore, plasma MIF can improve the diagnostic specificity for NPC patients combined with VCA-IgA [31], suggesting that high tumor-associated MIF expression may drive higher circulating levels of soluble MIF. Intracellular MIF can be stored in the cytosol or secreted into the extracellular space. MIF affects both tumor progression and tumor-associated immune responses. It is becoming increasingly evident that MIF plays an important regulatory role in governing TAM-dependent tumor initiation, progression, and metastatic disease phenotypes, although a unifying mechanism that explains how MIF contributes to this seemingly divergent M1 and M2 macrophage phenotypes is still lacking [32].

We found more CD68(+) macrophages in the tumor stroma than in the tumor nest, as well as in the non-epithelial areas of inflammation cases. Interestingly, more M1 (CD11c(+)) macrophages infiltrated the tumor nest than in the tumor stroma, and more M2 (CD163(+)) macrophages infiltrated the tumor stroma than the tumor nest. Huang et al. also reported that M2 macrophages presented high density in NPC stroma, which may be caused by the mesenchymal cells near the tumor nest attracting macrophages by generating chemotactic activity [33]. It is well known that macrophages tend to accumulate in hypoxic tumor areas, and tumor cells can release cytokines to switch macrophages to M2 macrophages, which can promote tumor progression [34]. Additionally, different levels of TAMs have been reported to have a different association with prognostic parameters [35]. A recent meta-analysis demonstrated that higher CD68(+) TAMs in tumor nests predicted favorable disease-free survival, and higher M2 (CD163(+)) macrophages were associated with poor survival outcomes in NPC [36]. Considering the promotion effect of M2 macrophages on tumors by producing vascular endothelial growth factor and extracellular matrix remodeling proteins, it is well understood that high M2 macrophages indicate a poor prognosis [37]. Ohri et al. found that more M1 macrophages in tumor nests were associated with a better prognosis in non-small cell lung cancer [38]. Although there was no information on prognosis in this study, HPV-related M1 macrophage infiltration in the tumor nest may have some effects on the prognosis of NPC patients with co-infection.

MIF has been reported to influence macrophage polarization. In glioma malignancy, MIF can inhibit M1 macrophages [39]. MIF also contributes to TAM polarization to the M2 subtype in tumor-bearing mice [40]. In our study, we evaluated the correlation between MIF expression levels and TAM-associated marker levels (CD68, CD11c, and CD163); however, there was no significant correlation between them. In contrast, we found a significant positive correlation between EBER1 levels and MIF expression levels in tumor nests. This suggests that EBV may promote MIF production in the tumor nest, affecting the progression of NPC. Few studies have examined the relationship between MIF and EBV, and more research is needed to understand the relationship between them. We also found a weak positive correlation between HPV16/18 level and the score for CD11c(+) cells in the tumor nest, indicating that there may be an interaction between HPV and M1 macrophages in NPC.

There are still several limitations that should be acknowledged. First, the patient’s clinical information is incomplete, which makes it impossible to analyze the relationship between these markers and the patient’s clinical prognosis. Second, in some cases, tissue cores were damaged and sample size reduced. Third is that the sample size was insufficient for analyzing HPV influence. Although we found a significant positive correlation between HPV16/18 and CD11c in NPC tumor nest, there were only 8 cases of HPV positive, which makes the correlation coefficient low. Due to these limitations, the samples with clinical information should increase in future study.

## Conclusions

This study is the first to investigate the relationship between EBV and HPV infection with MIF and TAM polarization in the same NPC tissue samples. ISH detected EBV(+) in 90% of NPC cases, including 10% EBV/HPV co-infection. A significant positive correlation between EBER1 levels and MIF expression levels in tumor nests and a significant positive correlation between HPV16/18 level and the score for CD11c(+) cells suggested the influence of viral infection on macrophage behavior.

## Supplementary Information

**Additional file 1: Figure S1**.

**Additional file 2: Figure S2**.

**Additional file 3: Figure S3**.

**Additional file 4: Figure S4**.

**Additional file 5: Figure S5**.

**Additional file 6: Figure S6**.

**Additional file 7: Table S1**.

## Data Availability

The datasets used and/or analyzed during the current study are available from the corresponding author on reasonable request. The gene expression profiles of MIF were downloaded from the Gene Expression Omnibus (GEO) (http://www.ncbi.nlm.nih.gov/geo/), accession numbers: GSE13597, GSE12452, and GSE53819. The public access to the databases is open.

## Abbreviations

EBV: Epstein–Barr virus
HPV: Human papillomavirus
MIF: Macrophage migration inhibitory factor
NPC: Nasopharyngeal carcinoma
NNE: Normal nasopharyngeal epithelium
TAMs: Tumor-associated macrophages
IL: Interleukin
BER: EBV-encoded RNA
FFPE: Formalin-fixed paraffin-embedded
ISH: In situ hybridization
IF: Immunofluorescence
GEO: The gene expression omnibus
