# Supplementary figures and images for: Influence of Epstein–Barr virus and human papillomavirus infection on macrophage migration inhibitory factor and macrophage polarization in nasopharyngeal carcinoma
Source: BMC Cancer. 2021 Aug 18;21:929. doi: 10.1186/s12885-021-08675-x (PMC8371777; doi:10.1186/s12885-021-08675-x)

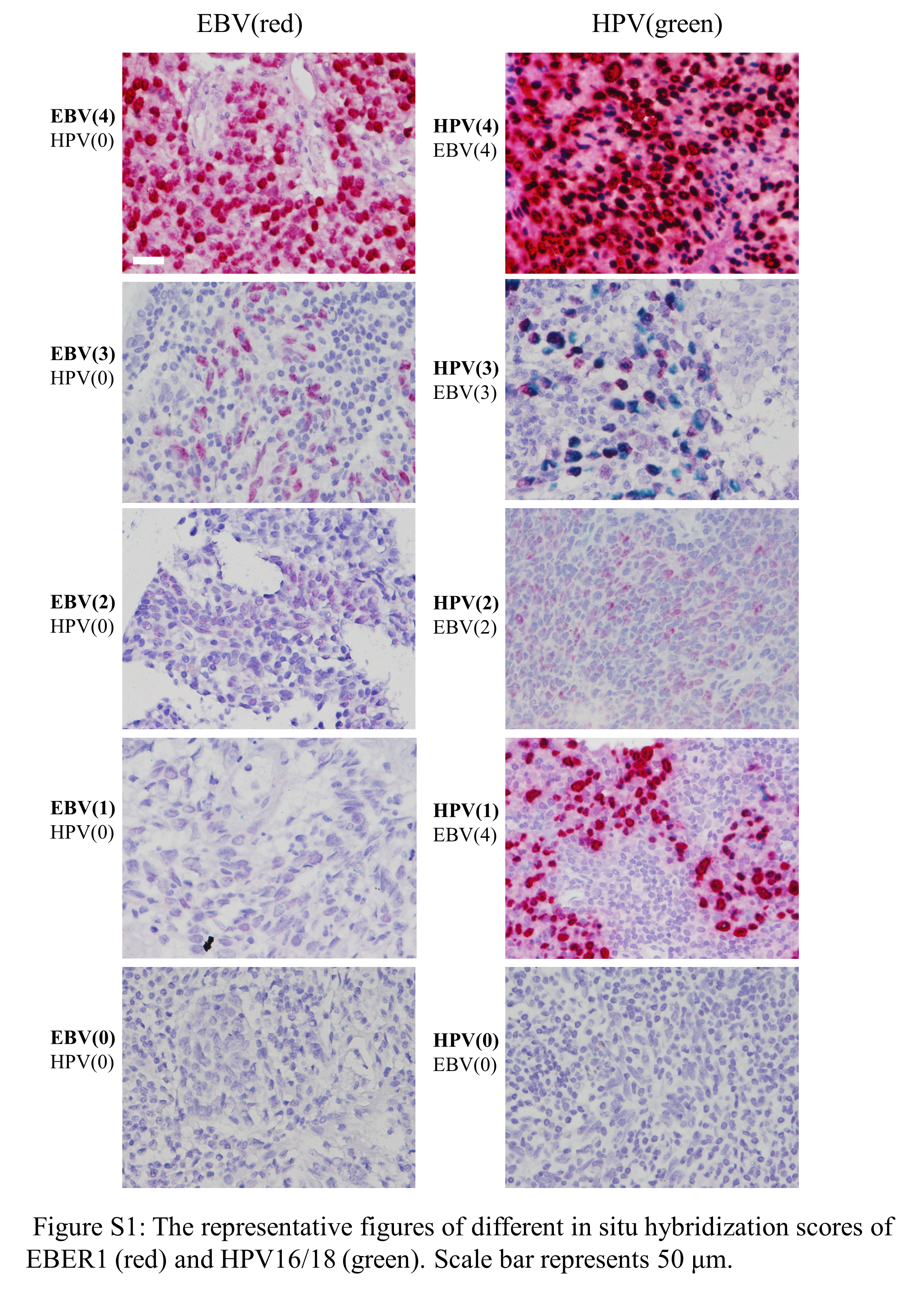

Supplement: Supplementary file 1 — Additional file 1: Figure S1. [file 12885_2021_8675_MOESM1_ESM.tif]

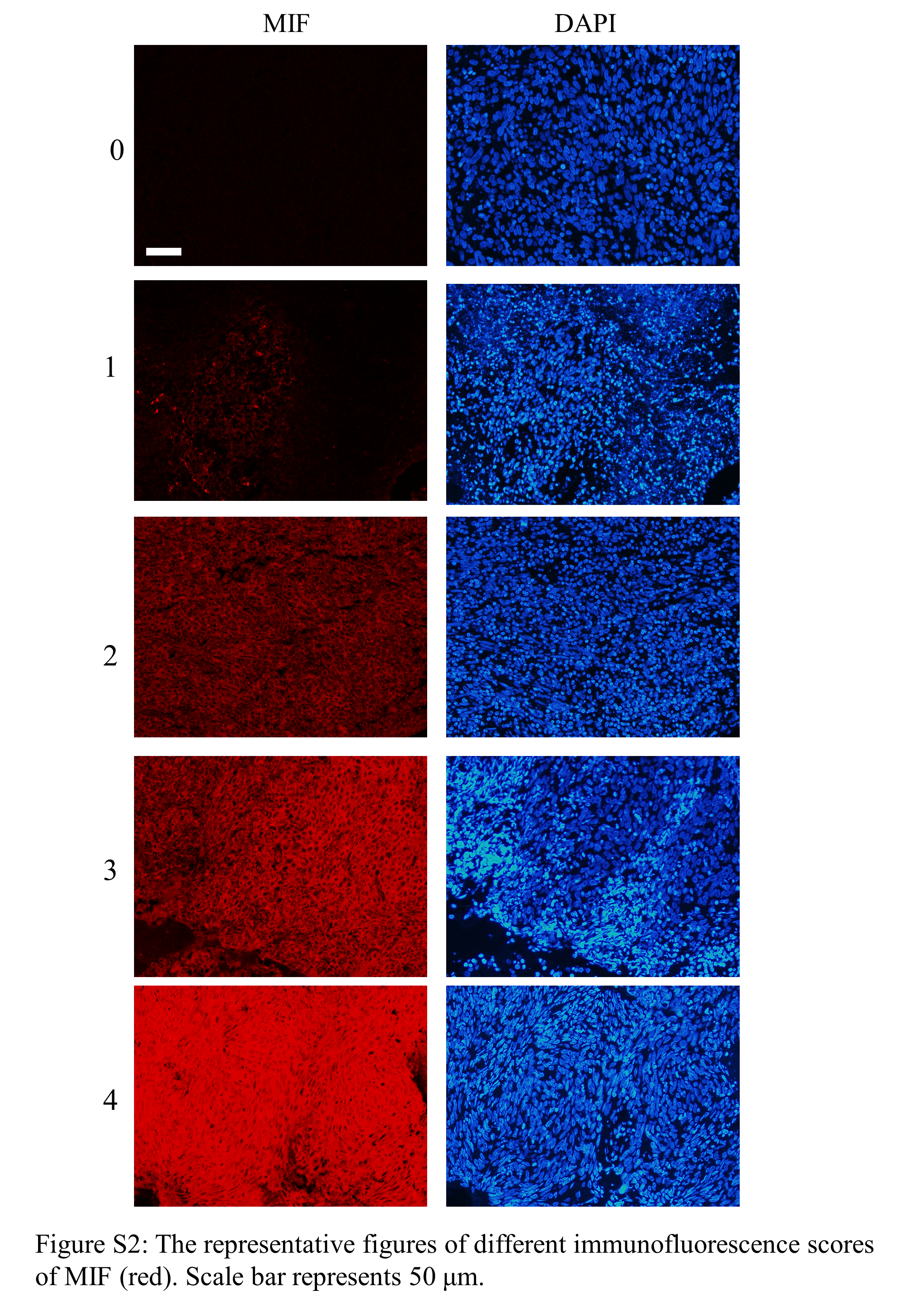

Supplement: Supplementary file 2 — Additional file 2: Figure S2. [file 12885_2021_8675_MOESM2_ESM.tif]

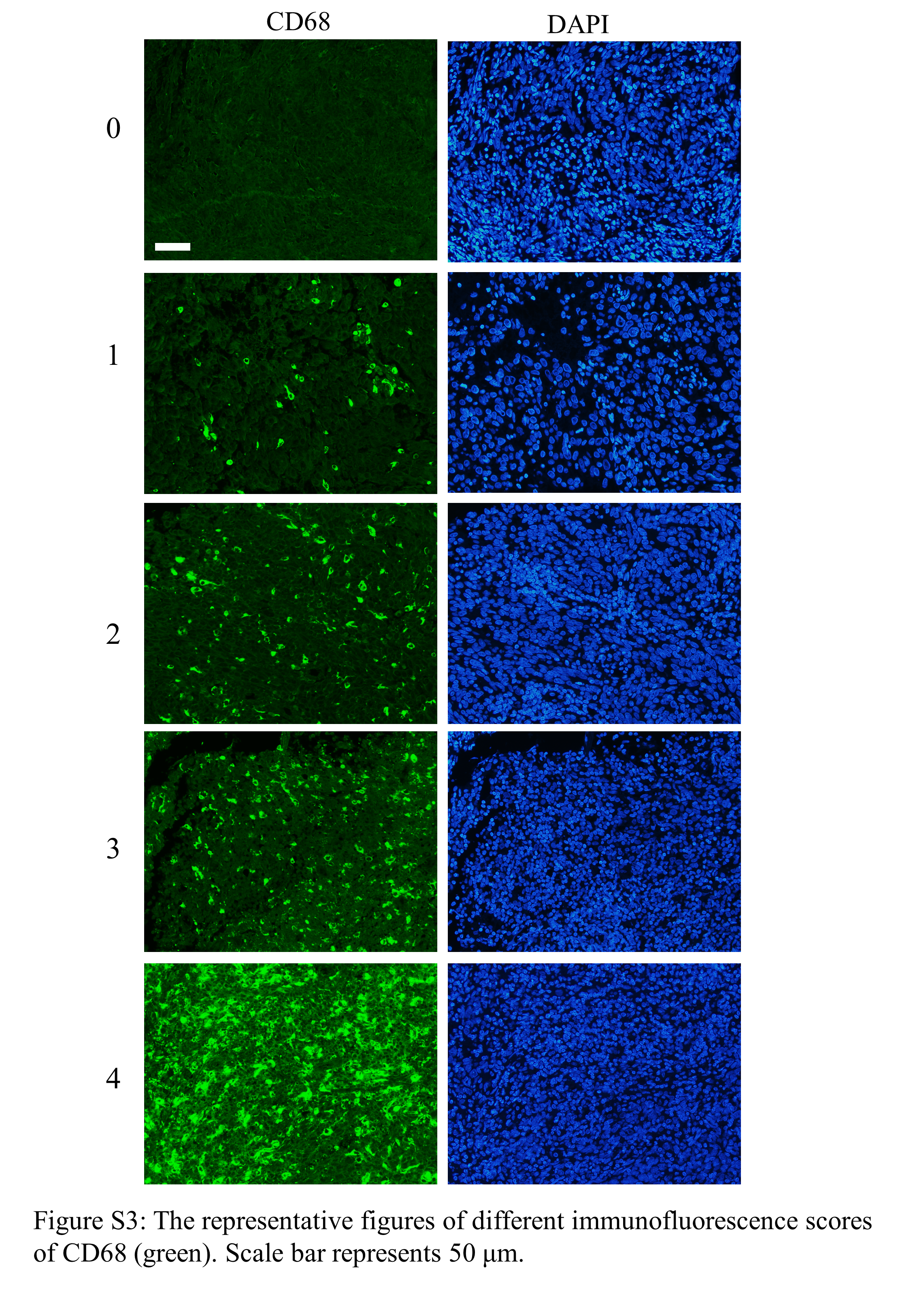

Supplement: Supplementary file 3 — Additional file 3: Figure S3. [file 12885_2021_8675_MOESM3_ESM.tif]

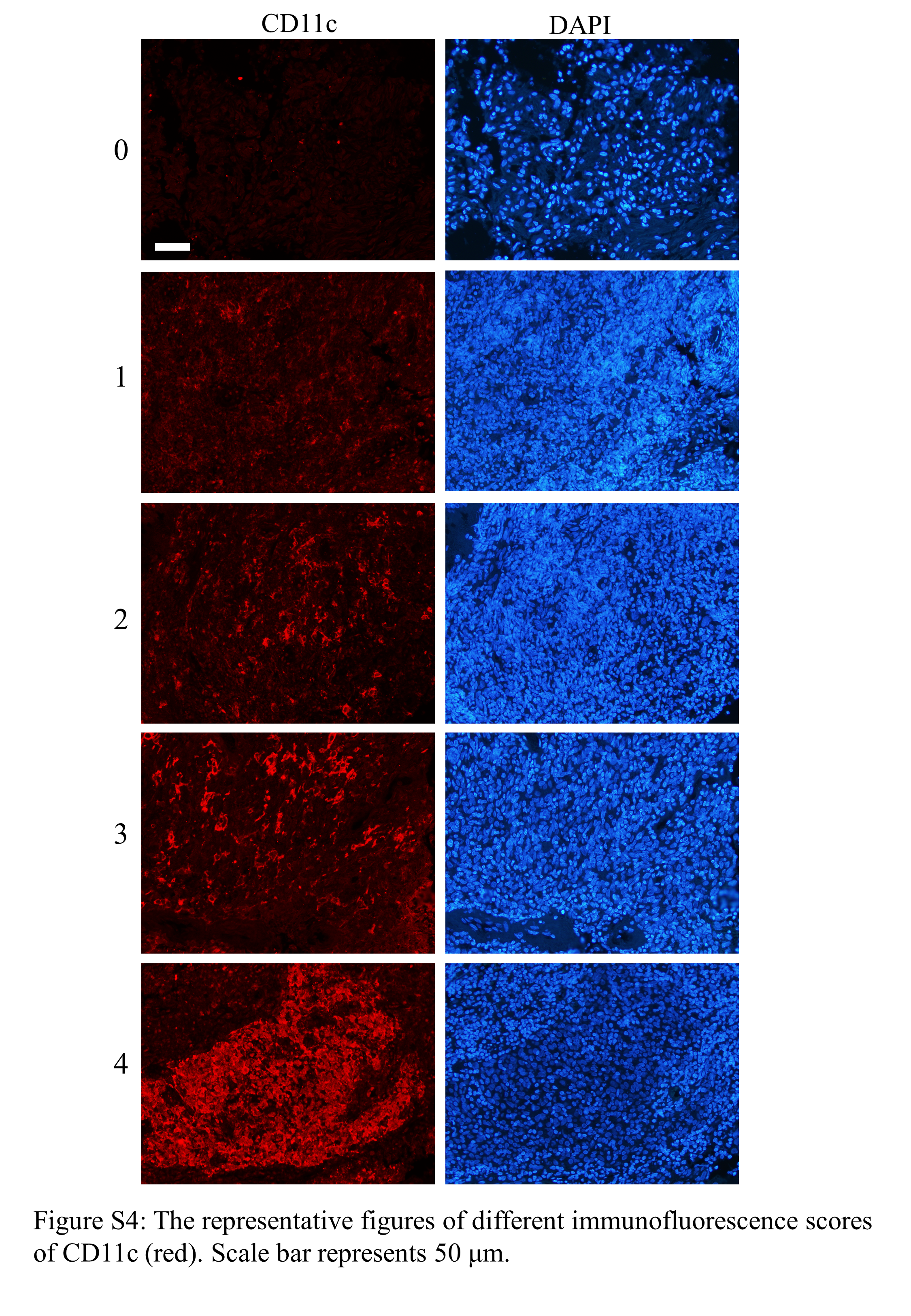

Supplement: Supplementary file 4 — Additional file 4: Figure S4. [file 12885_2021_8675_MOESM4_ESM.tif]

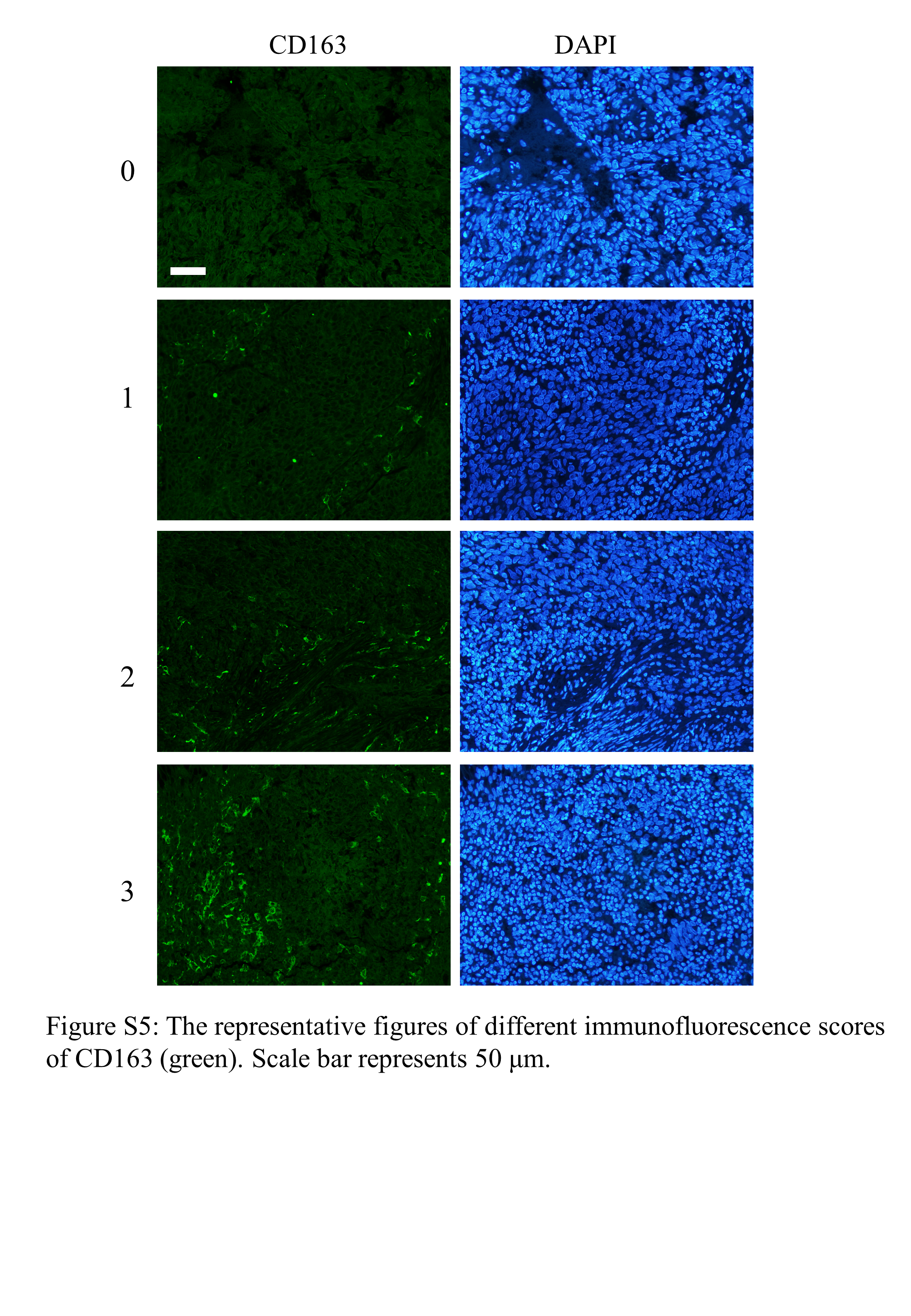

Supplement: Supplementary file 5 — Additional file 5: Figure S5. [file 12885_2021_8675_MOESM5_ESM.tif]

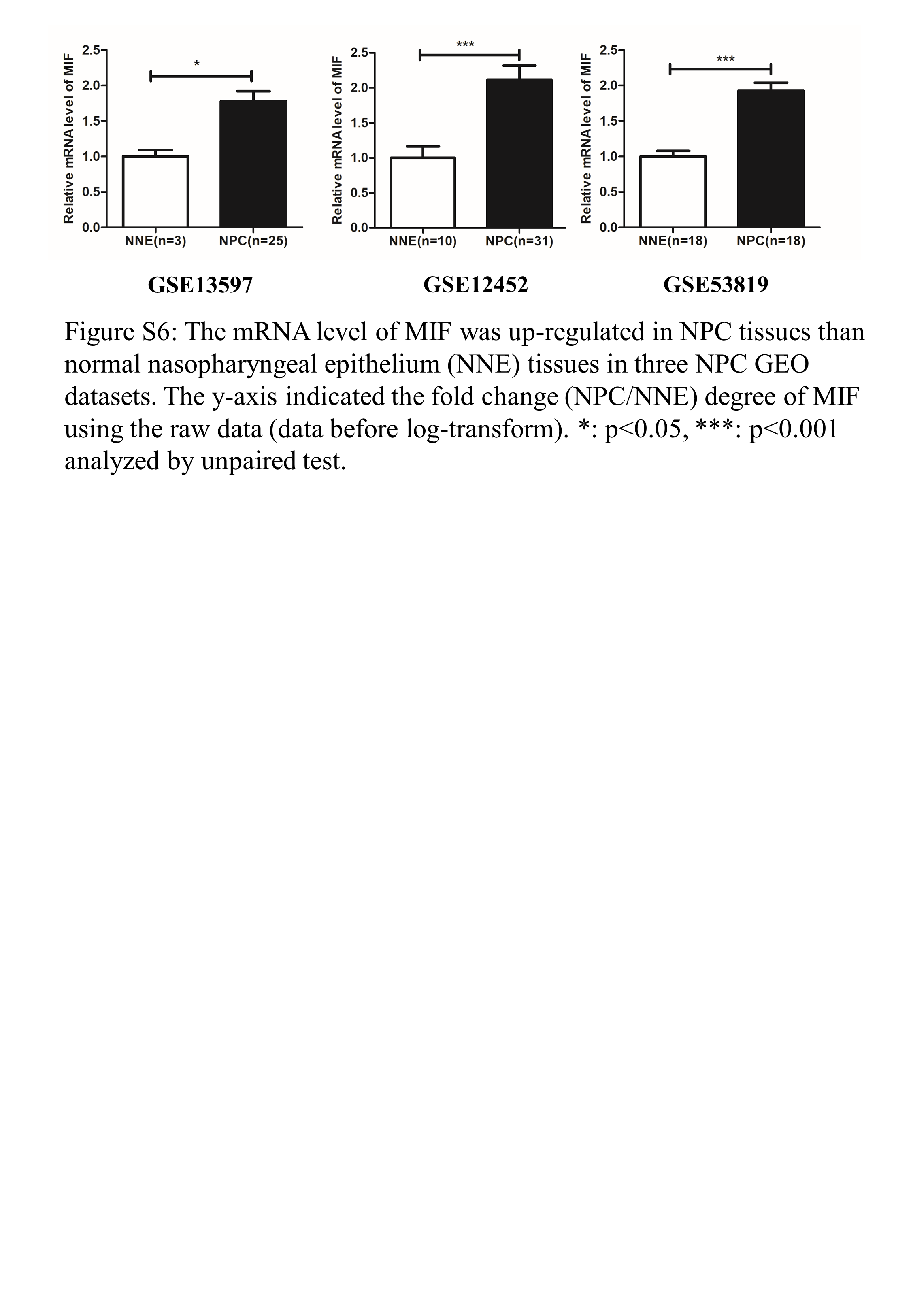

Supplement: Supplementary file 6 — Additional file 6: Figure S6. [file 12885_2021_8675_MOESM6_ESM.tif]
