# Supplementary material for: Influence of Epstein–Barr virus and human papillomavirus infection on macrophage migration inhibitory factor and macrophage polarization in nasopharyngeal carcinoma
Source: BMC Cancer. 2021 Aug 18;21:929. doi: 10.1186/s12885-021-08675-x (PMC8371777; doi:10.1186/s12885-021-08675-x)
Supplement: Supplementary file 7 — Additional file 7: Table S1. [file 12885_2021_8675_MOESM7_ESM.docx]

Additional file 7

Table S1. Correlation analysis between macrophage migration inhibitory factor (MIF) and macrophage markers in NPC.

| **Spearman**  **correlation** | |  | **CD68** | | **CD11c** | | **CD163** | |
| --- | --- | --- | --- | --- | --- | --- | --- | --- |
|  |  |  | **TN** | **TS** | **TN** | **TS** | **TN** | **TS** |
| **MIF** | **TN** | r | -0.117 | -0.176 | -0.055 | -0.068 | 0.043 | 0.039 |
|  |  | p | 0.312 | 0.126 | 0.663 | 0.593 | 0.729 | 0.754 |
|  |  | N | 77 | 77 | 65 | 65 | 66 | 66 |
|  | **TS** | r | -0.01 | -0.073 | 0.027 | -0.001 | 0.143 | 0.145 |
|  |  | p | 0.931 | 0.528 | 0.831 | 0.992 | 0.251 | 0.246 |
|  |  | N | 77 | 77 | 65 | 65 | 66 | 66 |

TN: tumor nest, TS: tumor stroma
